# Fluctuations in Maternal Depressive Symptoms, Anxiety, and Anger and Children’s Depression Risks in Middle Childhood

**DOI:** 10.1007/s10802-024-01201-0

**Published:** 2024-04-23

**Authors:** Qiong Wu

**Affiliations:** Department of Human Development & Family Science, College of Education, Health, and Human Sciences, Florida State University, Sandels 322, 120 Convocation Way, Tallahassee, FL USA

**Keywords:** Maternal depressive symptoms, Internalizing psychopathology, Environmental unpredictability, Anxiety, Anger, Affect dysregulation

## Abstract

Research suggests a robust link between the severity of maternal depression and children’s depression risks in middle childhood. Variations among depressed mothers in terms of affective dysregulation and frequent mood changes are also observed. However, the understanding of how fluctuations in maternal depressive symptoms and negative affect influence children is limited. Guided by life history theory, the current study tested whether the degree of fluctuations in maternal depressive symptoms, anxiety, and anger contributed to depression risks among school-aged children. The sample included 1,364 families where maternal depressive symptoms, anxiety, and anger were longitudinally assessed when children were in Grades 1, 3, 5, and 6. Children’s anxious depression and withdrawn depression behaviors were rated in Grades 1, 3, 4, 5, and 6 by two caregivers. Parallel latent growth curve analyses revealed that, first, fluctuations in maternal anxiety from Grade 1 to 6 were related to an increase in children’s withdrawn depression over the same period. Second, mean maternal anger over time was related to higher mean levels of child anxious and withdrawn depression, yet fluctuations in maternal anger were not linked to child outcomes. Findings support life history theory by highlighting the degree of fluctuations in maternal anxiety as a source of environmental unpredictability and reveal different effects of maternal anxiety and anger in the intergenerational transmission of depression, with important theoretical and clinical implications.

Maternal depression stands out as the most significant mental health concern for women of childbearing and child-raising ages (Meaney, 2018). Whereas some mothers experience a reduction in depressive symptoms over time, others face fluctuations and prolonged symptoms that can persist for years (Slomian et al., 2019). The prevalence and effects of maternal depression is noteworthy, given its potential impact on maternal parenting behaviors and subsequently, the heightened risk of depression in children. Offsprings of depressed mothers are more prone to emotional difficulties, with increased chances of lower emotion regulation and elevated behavioral problems, ultimately raising the risks of future psychiatric disorders such as depression and anxiety (Goodman et al., 2011; Slomian et al., 2019; Sutherland et al., 2022).

Decades of research suggest a robust association between the severity of maternal depression and children’s less optimal emotional development and risks for future depression (Slomian et al., 2019; Sutherland et al., 2022). Parenting literature also suggests that sub-clinically elevated depressive symptoms in mothers also affect their parenting behaviors and children’s mental health development (Dix et al., 2014; Premo & Kiel, 2016; Wu et al., 2019). Nevertheless, throughout child development, symptoms of maternal depression can change or fluctuate, but the understanding of how such *fluctuations* affect children accordingly is relatively limited. In addition, recent research suggests variations among depressed mothers in terms of their affective expressions, the frequency of mood changes, and the duration of depressive episodes (e.g., Field, 2010; Hooper et al., 2015; Wang & Dix, 2013). The current study sought to fill the research gaps by testing how fluctuations in maternal depressive symptoms and negative affect (anxiety and anger) were linked with the risks of depression in children during middle childhood by examining two possible socio-affective pathways, as follows.

## An Unpredictable Developmental Context

Theoretical models suggest a possibility that maternal depression introduces an unpredictable developmental setting for offspring (Goodman, 2007; Tronick & Reck, 2009). This unpredictability can be, in part, attributed to fluctuations in depressive symptoms over time. Depressed individuals may struggle to fulfill family responsibilities, encompassing day-to-day parenting activities, and may exhibit unpredictability in responding to children's emotions (Caughy et al., 2009; Kujawa et al., 2014; Silk et al., 2011; Wu et al., 2019).

In a context of unpredictability, *life history theory* provides theoretical support and elucidates how individuals strategically manage their limited resources based on their life experiences (Stearns, 1992). The harshness-unpredictability framework suggests that resource allocation may be influenced by environmental *harshness*, with individuals opting to mature faster in response to higher rates of adverse events (i.e., a higher environmental harshness level). However, challenges in learning and adapting to an *unpredictable* environment, characterized by stochastic variations in life-history conditions, can also arise and affect how individuals strategize to survive (Ellis et al., 2009; Young et al., 2020). An individual may use these two indicators (i.e., environmental harshness and unpredictability) jointly to learn about the environment in key developmental stages and formulate their unique life strategies to survive.

Young et al. (2020) proposed further conceptualization and measurement of environmental unpredictability. An individual may track the statistical features of the environment by continuous sampling and making sense of their lived experiences (i.e., a statistical learning perspective). They may then build a statistical model of the environment to predict and effectively respond to changes. The statistical model can include information on the overall (i.e., mean) level of harshness or any predictable trend (i.e., slope). However, there are almost always variations that are unpredictable, stochastic, or random in one’s environment; the degree of such variations may differ among individuals. Consistent with Young et al. (2020), this study operationalized unpredictability from a statistical learning perspective by analyzing stochastic variations within one’s life history. This approach is more congruent with the life history theory by indicating random variations or fluctuations in the contexts where individuals may collect information to learn about the environment and make effective life-strategy choices (Ellis et al., 2009, 2022; Young et al., 2020).

Young et al.’s (2020) framework separates the environmental features that individuals use to make life-strategy choices into the overall level (the mean), predictable changes (the slope), and unpredictable variations (fluctuations). Coincidentally, two aspects within longitudinal assessments are commonly evaluated to determine environmental features: the average level over time (the *mean*) and the rate of linear change (the *slope*; exemplary applications in the field of maternal depression include Gartstein et al., 2010; Matijasevich et al., 2015; van Der Waerden et al., 2015; Wu, 2021). However, the examination of fluctuations within maternal symptoms over time has been limited. Meanwhile, the large body of current research on life history theory operationalizes environmental unpredictability in terms of family income, neighborhood environment, and available resources that meet basic needs (e.g., Chen & Qu, 2017; Lam et al., 2022; Zhang et al., 2022). Nevertheless, the focus on maternal mental health as a source of environmental unpredictability has been insufficient. Conceptually, fluctuations in maternal depression and negative affect may increase maternal maladaptive caregiving behaviors and interrupt children’s emotion-related learning, thereby increasing the risk of depression (Granat et al., 2017; Gross et al., 2009; Kujawa et al., 2014; Wu, 2023). This is likely because children are too overwhelmed by variations in maternal mood and affective changes to learn about these emotions and derive effective coping strategies. Addressing these research gaps, the current study specifically focused on how fluctuations in maternal symptoms and negative affect influence their children.

Although limited evidence has explored the effects of fluctuations in maternal depressive symptoms over time on children, one recent study used two large, independent samples to demonstrate that maternal depressive symptoms during early childhood increased the depression risks among children (Wu, 2023). This effect was particularly prominent for mothers who were recovering from severe postpartum depression. Studies on maternal depression fluctuations during middle childhood have been few; one study indicated that one-time elevations of maternal symptoms, beyond the average levels, correlated with higher child internalizing behaviors during school transitions (Yan et al., 2021). These findings suggest that fluctuations in maternal depressive symptoms may pose an additional risk factor for children's depression, beyond the average levels.

## Dysregulated Negative Affect

Psychopathology theories propose that individuals with elevated depressive symptoms often exhibit dysregulated emotions, demonstrating either flattened affect or heightened levels of anger or anxiety (Joormann & Stanton, 2016; Rottenberg & Vaughan, 2008; Vanderlind et al., 2020). This dysregulation is associated with a diminished ability to cope with adverse events on a day-to-day basis, contributing to the fluctuations observed in depressive states over time (Hankin et al., 2005). Simultaneously, the current literature highlights the heterogeneity within depressed mothers. Various subtypes of depressed mothers have been identified, including those enduring chronic stress with persistent flat affect and anhedonia symptoms, those facing acute stressors with heightened negativity, anxiety, and dysphoria symptoms, and those experiencing a combination of both (Caughy et al., 2009; Hooper et al., 2015; Premo & Kiel, 2016; Silk et al., 2011; Wang & Dix, 2013). This heterogeneity extends to maternal parenting behaviors, resulting in two dominant parenting styles among depressed mothers: withdrawal coupled with a display of flattened affect or high negativity paired with overstimulation (Field, 2010; Goodman et al., 2020; Wu, 2021). The current evidence suggests a possibility that high levels of anxiety and anger may accompany maternal depression and may show fluctuations along with changes in depressive symptoms.

### Anxiety

Anxiety is the most common comorbid condition for women experiencing depression (Kessler et al., 2005). Anxiety and depression share similar affective, cognitive, and behavioral processes (Doom et al., 2021). Even so, the role of maternal anxiety in the intergenerational transmission of depression-related risks has been overlooked. However, the literature suggests a possibility that the intergenerational transmission of internalizing psychopathology is largely transdiagnostic (i.e., not diagnosis-specific, such as from maternal depression to child depression, but rather from maternal depression/anxiety to child depression/anxiety comorbidity; Doom et al., 2021; Starr et al., 2014). For example, a meta-analytic study supported the transdiagnostic transmission by showing that parental anxiety disorders increased the risks of both anxiety and depression among their offspring, although the risk was greater for offspring anxiety than for depressive disorders (Lawrence et al., 2019). However, an earlier meta-analysis suggested that parental anxiety was more likely to strictly relate to child anxiety-related disorders (i.e., diagnosis-specific), whereas the transmission between parental depressive and bipolar disorders to children was more likely to be transdiagnostic (Van Santvoort et al., 2015). When reviewing the current evidence, it is worth noting that not many studies have controlled for maternal depression when examining the effects of maternal anxiety on children. This lack of control reduces the validity of the transdiagnostic versus diagnosis-specific debate. In addition, studies specifically examining parental anxiety and childhood internalizing problems in middle childhood have been few, and most studies focus on a wider age range of children or the adolescence period (Lawrence et al., 2019).

### Anger

As an affect expression on the externalizing spectrum of psychopathology, anger expressions in depressed mothers are surprisingly common, possibly due to stress and difficulties in coping (Field et al., 2005; Vliegen & Luyten, 2008). Depression may be conceptualized as inward aggression towards oneself, whereas anger channels aggression outwards. Anger can manifest as irritability, hostility, frustration, and resentment among depressed mothers (Ou & Hall, 2018). Despite the prevalence, only a handful of studies have examined maternal anger in the context of maternal depression. Current evidence, mainly focusing on the period of early childhood, suggests that high-anger mothers behaved very similarly to high-anxiety mothers when interacting with their infants, although infants of high-anger mothers were less prone to changes in maternal behaviors than those of high-anxiety mothers (Field et al., 2005). Both depression and anger predicted parental over-reactive parenting toward preschoolers (Leung & Slep, 2006), and depressed mothers may particularly mirror back anger expressions toward their anger-prone preschoolers (Dix et al., 2014; Wu et al., 2019). Nevertheless, more research on maternal anger in the transmission of depression risks is needed, particularly in middle childhood, when children have more agency to learn about and respond to maternal anger.

Together, the fluctuations of maternal depressive symptoms, in combination with dysregulated negative affect displays (e.g., anxiety and anger), constitute an unstable and unpredictable developmental environment for children, and can contribute to children’s *depression risks*. In middle childhood, children’s depression risks can be conceptualized and assessed as their internalizing problem behaviors (i.e., depressive, anxious, and withdrawn behaviors; e.g., Doom et al., 2021; Lawrence et al., 2019). For example, maternal depressive symptoms have been related to higher behavioral problems and depressive symptoms among school-aged children (e.g., Gross et al., 2008; Yan et al., 2021). Due to the common comorbidity of depressive and anxiety symptoms in middle childhood (Doom et al., 2021; Hallett et al., 2009), two common phenotypes of depression-related behaviors can be observed: *anxious depression* and *withdrawn depression*. The former presents itself as a combination of sadness, feelings of worthlessness and hopelessness, and worries, whereas the latter can be shown as a lack of interest, flat affect, shyness, and social withdrawal. These cover the core symptoms of depression (i.e., sadness or lack of interest, with cognitive, affective, and behavioral symptomologies), whereas they represent two key subtypes of depression-anxiety comorbidity.

In middle childhood, children are developing vastly regarding emotion-related skills. They are thus sensitive to parental influences in the forms of affective expressions to learn about and statistically sample the emotional atmosphere in the surroundings (Criss et al., 2016; Dix et al., 2014). They are also grasping a wider range of emotion regulation strategies regarding more differentiated negative emotions, such as anger and anxiety (Morris et al., 2011). The current body of literature suggests a strong connection between maternal depression and children’s depression risks in middle childhood (e.g., Dix et al., 2014; Gross et al., 2008). In comparison, our current understanding is insufficient concerning dysregulated affect among mothers with elevated depressive symptoms; what is even more limited are studies utilizing a statistical approach that quantify and thus can elucidate different aspects of negative affect (i.e., the overall level, the predictable trend, and the unpredictable time-to-time fluctuations). Responding to this gap, the current study aimed at investigating the links between fluctuations in maternal depressive symptoms, anxiety, and anger, and the risks of depression during middle childhood.

## The Current Study

Guided by life history theory (Ellis et al., 2009, 2022; Young et al., 2020), the current study was the first one to empirically test whether the degree of fluctuations in maternal depressive symptoms, anxiety, and anger were linked with depression risks among children in middle childhood. This study focused on whether fluctuations in maternal symptoms and affect conferred additional risks to children beyond two commonly assessed factors in longitudinal studies: the mean and the slope. It utilized a large, longitudinal sample that spanned over six years in middle childhood. Child depression risks were operationalized as anxious depression and withdrawn depression behaviors among school-aged children and measured five times within a 6-year period.

**Fig. 1:**
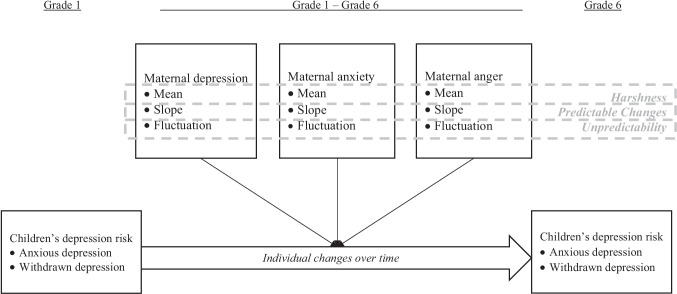
The conceptual model

Figure 1 presents the conceptual model of the current study. It was hypothesized that the degree of fluctuations in maternal depressive symptoms, in addition to the mean and the slope, would be related to depression risks among children (i.e., higher mean levels and faster increases in anxious depression and withdrawn depression behaviors over time). It was further expected that fluctuations in maternal anxiety and anger would link with higher depression risks among children beyond maternal depression.

## Method

### Participants

This study utilized data from the National Institute of Child Health and Human Development (NICHD) Study of Early Child Care and Youth Development (SECCYD), which included 1,364 participants, with 50.4% being boys. The participants consisted of infants born in 1991 from ten U.S. states. The mothers of these infants were over 18 years old, in good health, spoke English as their primary language, did not have the intention of putting their child up for adoption, resided within an hour of the research sites, and had no plans to relocate within the next three years (NICHD ECCRN, 2002). The average age of the mothers was 28.11 years (*SD* = 5.6). Most mothers (78.9%) identified as White, 11.2% as Black, 5.6% as Hispanic, and 4.3% as other. At the time of study enrollment, 85.2% of the mothers were cohabiting with their partners. The average length of maternal education was 14.4 years (*SD* = 2.5), and the average family income-to-needs ratio was 3.7 (*SD* = 2.8).

### Procedures

When children were as young as one-month-old, eligible mothers provided consent and parental permission for data collection. Mothers also provided demographic data. When children were in Grades 1, 3, 5, and 6 (referred to as G1, G3, G5, and G6 hereafter), data collection occurred where mothers self-rated their depressive symptoms, anger, and anxiety at each time point. Mothers and alternative caregivers (usually fathers) independently rated child problem behaviors at G1, G3, G4, G5, and G6. The involved university’s Institutional Review Board provided ethical approval.

### Measures

Maternal depressive symptoms at G1, G3, G5, and G6 were self-assessed using the Center for Epidemiological Study-Depression Inventory (CES-D; Radloff, 1977) to capture experienced symptoms over the past week, rated on a 4-point scale (0 = *less than 1 day*, 3 = *5–7 days*). The total score from the 20 items was utilized. The CES-D is a validated instrument designed for both clinical and non-clinical populations. Previous reliability and validation studies have demonstrated the CES-D's internal consistency (α > 0.90) and test-retest reliability (*r*s > 0.50; Vilagut et al., 2016; Yang et al., 2017). In the present sample, Cronbach's α ranged from 0.85 to 0.91 across time points.

Maternal anxiety and anger were self-rated on the 20-item State-Trait Anger & Anxiety Scales (Spielberger et al., 1983) at G1, G3, G5 and G6. The scales assess how respondents felt over the last week, including a 10-item state anxiety subscale and a 10-item state anger subscale. Each item was rated on a 4-point Likert scale (1 = *not at all in the past week* to 4 = *most or all of the time*). Example items include “I feel nervous” and “I am mad” for anxiety and anger, respectively. Higher scores indicated more symptoms. The measure has good internal consistency, ranging from 0.89 to 0.95 across time points.

Child anxious depression and withdrawn depression at G1, G3, G4, G5, and G6 were independently rated by the mother and one alternative caregiver (mainly, the father), with DSM-oriented anxious/depressed and withdrawn/depressed behaviors in the Child Behaviors Checklist (CBCL; ages 4–18; Achenbach, 1991). Children’s behaviors were rated on a 3-point scale (0 = *not true*, 2 = *very true*). Examples of the 13-item anxious/depressed subscale include “cries a lot” and “fears,” and those of the 8-item withdrawn/depressed subscale include “sad” and “shy, timid.” T-scores of these subscales were used, and an average score between two reporters was generated, with higher scores showing more elevated symptoms. The scales showed a satisfactory internal consistency (0.74–0.81 for anxious/depressed and 0.63–0.74 for withdrawn/depressed subscales, respectively).

Covariates included child sex, race, and birth order; maternal marital status, non-marital cohabitation, age, years of education, and family income-to-needs ratio, reported by mothers at 1-month postpartum. In addition, child negativity was reported by mothers using the Children's Behavior Questionnaire (CBQ; Rothbart et al., 1994) at 54 months old. A mean score was generated with the standardized scores of three 10-item subscales: sadness, fear, and anger (Cronbach's α ranged from 0.60 to 0.85). A higher score indicated higher negativity, which was used as a temperamental covariate in the analyses.

### Data Analytic Strategies

Analysis was conducted using the Mplus program. Preliminary data processing included the generation of a mean, a slope, and a within-person variation variable. The steps were demonstrated below using maternal depressive symptoms as an example. Following Wang et al. (2012), a linear longitudinal multilevel model (with an intercept, a slope regressed on time, and random effects at the within-individual and between-individual levels) was estimated with FIML in the Mplus program to generate the linear rate of change (i.e., the slope) in maternal depressive symptoms from G1 to G6 for each mother. Then, the “detrended” maternal depressive symptom scores for each mother from G1 to G6 were calculated, using the original symptom scores to subtract the rate of change multiplied by time at each time point. The detrended mean (referred to as the “mean” hereafter) was then calculated, by averaging the detrended data for each mother from G1 to G6, to provide a “true” mean without the effect of linear changes over time. The mathematical equation is as follows. X_*i*_ indicates the depressive symptom scores at time point *i* (from G1 to G6, for a maximum of four time points) for each mother; s_0_ indicates the slope for each mother generated from Mplus; X_*de_i*_ indicates the detrended depressive symptom scores at time point *i*; *n* indicates the number of time points (calculated for each mother deducting missed assessment points); and X_*mean*_ indicates the detrended mean for each mother.

$${X}_{de\_i}={X}_{i}-{s}_{0}*i$$

$${X}_{mean}= \frac{\sum_{i=1}^{n}{X}_{de\_i}}{n}$$

Next, an intraindividual standard deviation (*ISD*) was computed with detrended depressive symptom data from G1 to G6 to indicate the extent of variation or fluctuation in depressive symptoms over time beyond the mean and the slope, as follows.

$$ISD=\sqrt{\frac{\sum_{i=1}^{n}{({X}_{de\_i}-{X}_{mean})}^{2}}{n-1}}$$

Statistically, this approach to deriving these variables is equivalent to a linear longitudinal multilevel model in estimating the mean and the linear slope. Within-individual variations after the mean and the slope for each individual were determined were calculated as fluctuations - in the multilevel model, these are deemed as random effects or residual variances. The current approach complements a traditional multilevel model. In a multilevel model, random effects are treated as prediction errors, which means it cannot utilize this information as a predictor, as done in this study.

A Parallel Latent Growth Curve Model was then estimated for child anxious depression and withdrawn depression behaviors to estimate individual means and slopes from G1 to G6. The mean, the slope, and the *ISD* of maternal depressive symptoms, anxiety, and anger were used as predictors towards intercepts and slopes of child anxious depression and withdrawn depression behaviors, along with other covariates. The model fit was evaluated by root mean squared error of approximation (RMSEA; < 0.05), comparative fit index (CFI; > 0.95), and the Tucker–Lewis index (TLI; > 0.95).

Little’s MCAR test (Little, 1988) revealed that data were not missing completely at random, χ^2^(1450) = 2172.62, *p* < 0.001. Missingness in maternal depression, anxiety, and anger ratings were related to a higher birth order, mother not married, not cohabiting, younger in age, lower education levels, and lower income-to-needs ratios, *t*s ≥ 2.6, *p*s ≤ 0.02. Missingness in child problem behaviors from G1 to G6 was related to the child being male, mother not married, younger in age, and having a lower income-to-needs ratio; missingness in child problem behaviors at G1 was additionally related to the child being racially minority, a higher birth order, and mother not cohabiting, *t*s ≥ 2.1, *p*s ≤ 0.03. As missingness of the key study variables can be explained by demographic covariates, the missing information can be at least recoverable with these variables controlled for in the model. Full information likelihood (FIML) estimation was then used to reduce bias when estimating the missing information (Enders & Bandalos, 2001).

## Results

Descriptive statistics of variables are presented in Table 1. On a bivariate level, the mean and the *ISV*s of maternal depressive symptoms, anxiety, and anger were related to higher child anxious depression and withdrawn depression behaviors. The slope of maternal depressive symptoms and maternal anger were associated with relatively few child anxious depression and withdrawn depression behaviors across time points.

**Table 1:** Descriptive statistics and bivariate correlation for study variables

	1	2	3	4	5	6	7	8	9	10	11	12	13	14
1. Child Sex														
2. Child Race	-0.02													
3. Child Birth Order	0.01	-0.08**												
4. Married	-0.02	0.41**	-0.01											
5. Cohabitation	-0.01	0.35**	0.03	0.74**										
6. Mother's Age	0.03	0.26**	0.22**	0.43**	0.34**									
7. Mother's Education	0.04	0.20**	-0.12**	0.40**	0.31**	0.55**								
8. Income-to-Needs Ratio 1 M	0.01	0.20**	-0.12**	0.34**	0.28**	0.39**	0.42**							
9. Child Negativity	0.02	0.00	-0.04	-0.05	0.00	-0.04	-0.01	-0.02						
10. Depression Mean	0.01	-0.14**	0.05	-0.22**	-0.20**	-0.19**	-0.25**	-0.18**	0.24**					
11. Depression Slope	0.02	-0.03	0.00	0.02	0.02	-0.04	0.02	-0.05	0.01	0.00				
12. Depression ISV	0.00	-0.06	0.04	-0.15**	-0.12**	-0.13**	-0.15**	-0.09**	0.13**	0.60**	0.08*			
13. Anxiety Mean	0.01	-0.10**	0.02	-0.15**	-0.14**	-0.14**	-0.17**	-0.13**	0.27**	0.82**	0.05	0.49**		
14. Anxiety Slope	0.00	-0.03	-0.01	-0.08*	-0.08*	-0.05	-0.05	-0.03	0.11**	0.22**	0.66**	0.19**	0.28**	
15. Anxiety ISV	-0.04	0.02	0.02	-0.07*	-0.06*	-0.07*	-0.06	-0.05	0.10**	0.28**	0.02	0.52**	0.33**	0.14**
16. Anger Mean	0.01	-0.05	0.01	-0.14**	-0.12**	-0.13**	-0.16**	-0.12**	0.20**	0.69**	0.00	0.38**	0.66**	0.13**
17. Anger Slope	-0.05	-0.03	-0.01	-0.03	-0.05	-0.07*	-0.04	-0.02	0.03	0.08**	0.52**	0.08**	0.07*	0.52**
18. Anger ISV	-0.02	-0.07*	0.02	-0.16**	-0.15**	-0.21**	-0.21**	-0.13**	0.12**	0.43**	0.05	0.46**	0.43**	0.13**
19. Anxious Depression G1	-0.02	-0.01	-0.11**	-0.04	-0.01	-0.11**	-0.06*	0.02	0.29**	0.24**	-0.05	0.11**	0.25**	0.04
20. Anxious Depression G3	-0.01	0.02	-0.10**	-0.06	-0.03	-0.06*	-0.04	0.01	0.25**	0.33**	-0.01	0.17**	0.31**	0.10**
21. Anxious Depression G4	-0.04	0.03	-0.05	-0.03	0.01	-0.11**	-0.07*	-0.07*	0.20**	0.30**	0.02	0.14**	0.29**	0.11**
22. Anxious Depression G5	-0.02	0.02	-0.04	-0.01	-0.01	-0.10**	-0.04	-0.04	0.20**	0.31**	0.06	0.19**	0.32**	0.13**
23. Anxious Depression G6	-0.07*	0.01	-0.03	-0.02	0.01	-0.06*	-0.03	-0.05	0.20**	0.28**	0.04	0.18**	0.26**	0.13**
24. Withdrawn Depression G1	-0.07*	-0.08*	-0.04	-0.07*	-0.06	-0.13**	-0.09**	-0.03	0.16**	0.22**	-0.05	0.12**	0.21**	0.00
25. Withdrawn Depression G3	-0.07*	-0.05	-0.03	-0.06*	-0.07*	-0.10**	-0.07*	-0.07*	0.17**	0.29**	-0.02	0.17**	0.24**	0.06
26. Withdrawn Depression G4	-0.06	-0.06	-0.05	-0.08*	-0.06	-0.17**	-0.13**	-0.15**	0.08*	0.24**	0.02	0.13**	0.21**	0.07*
27. Withdrawn Depression G5	-0.07*	-0.05	-0.06	-0.06	-0.06*	-0.16**	-0.10**	-0.09**	0.12**	0.28**	0.04	0.16**	0.28**	0.09**
28. Withdrawn Depression G6	-0.08**	-0.04	-0.04	-0.05	-0.02	-0.08**	-0.06	-0.09**	0.12**	0.28**	0.02	0.17**	0.25**	0.10**
*N*	1364	1364	1364	1362	1362	1364	1363	1273	1060	1125	1125	1074	1125	1125
Minimum	1.00	0.00	1.00	0.00	0.00	18.00	7.00	0.08	-3.07	-0.19	-1.47	0.01	10.00	-0.81
Maximum	2.00	1.00	7.00	1.00	1.00	46.00	21.00	25.08	2.19	48.46	2.10	21.49	35.45	0.75
Mean	–	–	1.83	–	–	28.11	14.23	2.86	-0.01	8.54	0.08	4.49	17.38	0.01
*SD*	–	–	0.95	–	–	5.63	2.51	2.61	0.80	6.95	0.33	3.42	4.08	0.17

	15	16	17	18	19	20	21	22	23	24	25	26	27	28
1. Child Sex														
2. Child Race														
3. Child Birth Order														
4. Married														
5. Cohabitation														
6. Mother's Age														
7. Mother's Education														
8. Income-to-Needs Ratio 1 M														
9. Child Negativity														
10. Depression Mean														
11. Depression Slope														
12. Depression ISV														
13. Anxiety Mean														
14. Anxiety Slope														
15. Anxiety ISV														
16. Anger Mean	0.25**													
17. Anger Slope	0.02	-0.04												
18. Anger ISV	0.45**	0.60**	0.08**											
19. Anxious Depression G1	0.07*	0.29**	-0.01	0.17**										
20. Anxious Depression G3	0.09**	0.33**	0.04	0.15**	0.57**									
21. Anxious Depression G4	0.11**	0.30**	0.05	0.15**	0.53**	0.70**								
22. Anxious Depression G5	0.13**	0.33**	0.02	0.19**	0.46**	0.64**	0.70**							
23. Anxious Depression G6	0.13**	0.27**	0.08*	0.13**	0.47**	0.61**	0.65**	0.73**						
24. Withdrawn Depression G1	0.05	0.24**	0.00	0.14**	0.57**	0.36**	0.36**	0.32**	0.30**					
25. Withdrawn Depression G3	0.11**	0.30**	0.01	0.14**	0.32**	0.59**	0.44**	0.39**	0.41**	0.52**				
26. Withdrawn Depression G4	0.12**	0.27**	0.04	0.18**	0.36**	0.46**	0.59**	0.48**	0.45**	0.53**	0.64**			
27. Withdrawn Depression G5	0.09**	0.29**	0.06	0.15**	0.35**	0.43**	0.48**	0.62**	0.46**	0.54**	0.57**	0.69**		
28. Withdrawn Depression G6	0.15**	0.28**	0.05	0.15**	0.34**	0.39**	0.44**	0.49**	0.66**	0.41**	0.54**	0.59**	0.61**	
*N*	1074	1125	1125	1074	1047	1030	1027	1021	1029	1047	1030	1027	1021	1029
Minimum	0.00	9.99	-1.63	0.01	50.00	50.00	50.00	50.00	50.00	50.00	50.00	50.00	50.00	50.00
Maximum	13.26	31.35	1.17	16.30	79.00	89.00	90.00	81.50	81.00	81.00	86.00	88.00	84.00	86.00
Mean	3.08	13.83	-0.01	2.28	52.80	53.22	52.84	52.96	52.82	52.77	52.80	52.71	52.91	52.67
*SD*	1.92	3.29	0.23	1.87	4.27	4.93	4.72	4.79	4.85	4.19	4.39	4.66	4.81	4.78

Child sex: 1 = *male*, 2 = *female*. Child race: 1 = *White,* 0 = *other*. Mother married: 1 = *married*, 0 = *not married*. Cohabitation: 1 = *cohabitating*, 0 = *not cohabitating*

*1 M* 1 month, *G1* Grade 6

*p < 0.05; **p < 0.01

An unconditional Parallel Latent Growth Curve Model was first estimated for child anxious depression and withdrawn depression behaviors from G1 to G6. This model showed an acceptable fit, χ^2^(36) = 165.16, *p* = 0.001; CFI = 0.98, TLI = 0.98, RMSEA = 0.06 (CI_90%_ [0.05, 0.07]). Over time, child anxious depression and withdrawn depression behaviors were stable (the slope for anxious depression = -0.02, *SE* = 0.03, *t* = -0.78, *p* = 0.44; the slope for withdrawn depression = -0.02, *SE* = 0.03, *t* = -0.59, *p* = 0.56). Both the intercepts and slopes for child anxious depression and withdrawn depression behaviors had significant variances (*p* < 0.001), laying a foundation to add predictors.

**Fig. 2:**
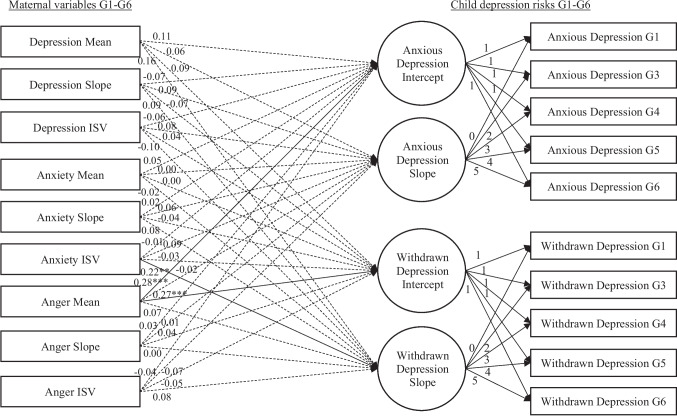
The analytic model. *Note:* Standardized coefficients are shown for the structural model. Covariance and covariates are omitted for simplicity. *p < 0.05; **p < 0.01; ***p < 0.001

The conditional model (with predictors; Table 2; Fig. 2) showed a good model fit, χ^2^(144) = 315.39, *p* = 0.001; CFI = 0.98, TLI = 0.96, RMSEA = 0.03 (CI_90%_ [0.03, 0.03]). The mean, slope, or *ISV* of maternal depressive symptoms were not related to intercepts or slopes of child anxious or withdrawn depression. The *ISV* of maternal anxiety was related to a faster increase in child withdrawn depression (*B* = 0.06, *SE* = 0.02, *t* = 3.06, *p* = 0.002). The mean of maternal anger was related to higher means of child anxious depression (*B* = 0.31, *SE* = 0.06, *t* = 5.07, *p* < 0.001) and withdrawn depression (*B* = 0.26, *SE* = 0.06, *t* = 4.38, *p* < 0.001). The slope or *ISV* of maternal anger was not related to the child's anxious or withdrawn depression.

**Table 2:** Model results

	Child Anxious Depression								Child Withdrawn Depression							
	Intercept R^2^ = 0.27				Slope R^2^ = 0.09				Intercept R^2^ = 0.18				Slope R^2^ = 0.13			
	*B*	*SE*	*t*	*β*	*B*	*SE*	*t*	*β*	*B*	*SE*	*t*	*β*	*B*	*SE*	*t*	*β*
Child Sex	-0.09	0.24	-0.36	-0.01	-0.06	0.06	-1.10	-0.05	**-0.52**	**0.23**	-2.23*	**-0.08**	-0.01	0.06	-0.25	-0.01
Child Race	0.23	0.35	0.66	0.03	0.06	0.08	0.72	0.04	-0.47	0.34	-1.39	-0.06	0.06	0.08	0.74	0.05
Child Birth Order	**-0.51**	**0.14**	-3.56***	**-0.13**	**0.09**	**0.03**	2.59**	**0.14**	-0.03	0.14	-0.22	-0.01	-0.04	0.03	-1.29	-0.08
Married	-0.21	0.47	-0.45	-0.03	0.12	0.11	1.03	0.08	0.49	0.47	1.05	0.06	0.01	0.11	0.13	0.01
Cohabitation	0.50	0.54	0.94	0.05	0.00	0.13	0.01	0.00	-0.42	0.53	-0.79	-0.05	0.11	0.13	0.87	0.08
Mother's Age	-0.04	0.03	-1.42	-0.06	0.00	0.01	-0.49	-0.03	**-0.08**	**0.03**	-2.64**	**-0.13**	0.00	0.01	0.57	0.04
Mother's Education	-0.03	0.06	-0.49	-0.02	0.02	0.02	1.35	0.08	-0.02	0.06	-0.30	-0.01	0.01	0.02	0.55	0.04
Income-to-Needs Ratio	0.10	0.06	1.66	0.07	**-0.03**	**0.01**	-2.27*	**-0.13**	0.07	0.05	1.26	0.06	**-0.04**	**0.01**	-3.09**	**-0.21**
Child Negativity	**1.16**	**0.17**	7.03***	**0.26**	**-0.09**	**0.04**	-2.16*	**-0.11**	**0.54**	**0.16**	3.33***	**0.13**	-0.07	0.04	-1.72	-0.11
Depression Mean	0.06	0.04	1.53	0.11	0.01	0.01	0.55	0.06	0.04	0.04	1.10	0.09	0.01	0.01	1.29	0.16
Depression Slope	-0.81	0.51	-1.57	-0.07	0.16	0.12	1.36	0.09	-0.64	0.50	-1.28	-0.07	0.13	0.12	1.09	0.09
Depression ISV	-0.06	0.05	-1.13	-0.06	0.01	0.01	1.13	0.08	0.04	0.05	0.69	0.04	-0.01	0.01	-1.18	-0.10
Anxiety Mean	0.05	0.06	0.85	0.05	0.00	0.01	-0.05	0.00	0.00	0.05	0.06	0.00	0.00	0.01	-0.16	-0.02
Anxiety Slope	0.31	1.01	0.31	0.02	0.20	0.24	0.84	0.06	-0.68	0.99	-0.69	-0.04	0.23	0.23	1.00	0.08
Anxiety ISV	-0.02	0.08	-0.21	-0.01	0.03	0.02	1.53	0.09	-0.06	0.08	-0.69	-0.03	**0.06**	**0.02**	3.06**	**0.22**
Anger Mean	**0.31**	**0.06**	5.07***	**0.28**	0.00	0.01	-0.24	-0.02	**0.26**	**0.06**	4.38***	**0.27**	0.01	0.01	0.75	0.07
Anger Slope	0.45	0.63	0.71	0.03	0.02	0.15	0.15	0.01	0.57	0.62	0.93	0.04	0.01	0.14	0.05	0.00
Anger ISV	-0.07	0.09	-0.74	-0.04	-0.02	0.02	-1.04	-0.07	-0.08	0.09	-0.89	-0.05	-0.02	0.02	-0.94	-0.08

Child sex: 1 = *male*, 2 = *female*. Child race: 1 = *White,* 0 = *other*. Mother married: 1 = *married*, 0 = *not married*. Cohabitation: 1 = *cohabitating*, 0 = *not cohabitating*

Bold texts indicate statistically significant findings

*1 M* 1 month, *G1* Grade 6

*p < 0.05; **p < 0.01; ***p < 0.001

## Discussion

Guided by life history theory (Ellis et al., 2009; Young et al., 2020), the current study examined whether the degree of fluctuations in maternal anxiety and anger, in the context of maternal depressive symptoms, was linked to depression risks among children in middle childhood. Findings suggested different functions of anxiety and anger in the context of maternal depressive symptoms, with implications for theorizing this topic.

This study found that a higher degree of fluctuations of maternal anxiety was linked to an increase in child withdrawn depression over time. This finding seems to suggest that fluctuations of maternal anxiety can serve as an unpredictable environment for children. When children encounter significant variations in maternal anxiety, they may be overburdened to learn about and cope with this emotion effectively (Lawrence et al., 2019). This effect may be particularly relevant in the context of maternal depression risk transmission, given the high comorbidity of depression and anxiety (Kessler et al., 2005). It is likely that when anxiety symptoms arise, depressive symptoms also intensify, as they share similar affective, cognitive, and behavioral mechanisms (Doom et al., 2021). Further, when depressed mothers experience anxiety, they may particularly show maladaptive parenting behaviors – either intrusive or withdrawn – and this may intensify risks in their children (e.g., Field et al., 2005; Granat et al., 2017). As a result of such variation in maladaptive parenting behaviors, their children can manifest behavioral symptoms such as a lack of affect expressions, socially withdrawn behaviors, and loss of motivation and interest (Caughy et al., 2009). This finding complements current evidence on life history theory (Ellis et al., 2009; Young et al., 2020) in considering fluctuations in maternal anxiety symptoms as a source of unpredictability in shaping children’s development.

As for anger, the mean of maternal anger was also linked to higher mean levels of child anxious depression and withdrawn depression. These findings complement previous studies focusing on younger samples (e.g., Field et al., 2005; Leung & Slep, 2006) by showing the average level of maternal anger as a predictor of children’s depression risks in middle childhood. It is worth noting that maternal anger was linked to both subtypes of children’s depression-anxiety comorbidity. It appears that maternal anger, as an aggressive emotion, may increase children’s fear, withdrawal, and inwardly directed aggression toward oneself, thus increasing children’s depression risks (Caughy et al., 2009; Leung & Slep, 2006; Silk et al., 2011).

Contrary to expectations, the fluctuation of maternal anger was not related to child outcomes. Likely, the overall level (or the mean) of maternal anger is a stronger predictor of child depression risks in comparison to its variation. It is also likely that some variations in anger expression may not be entirely undesirable for children. Past research also suggested that experiencing a healthy variation of maternal anger may help children recognize, understand, and regulate this emotion, as long as such expression is not overly excessive (Halberstadt & Eaton, 2002; Lunkenheimer et al., 2011; Wu et al., 2019). This may be particularly important in the context of maternal depression, as depressed mothers can be relatively rigid and less flexible in their affective responses to their children (Dix et al., 2014; Premo & Kiel, 2016; Wu et al., 2019). Experiencing a spectrum of anger intensity from their mothers who are dealing with depression may provide children with opportunities to normalize this emotion, gain insights into the diverse consequences of their actions, and establish adaptable interpersonal behaviors and boundaries. Conversely, in cases where depressed mothers consistently express anger rigidly, their children may tend to perceive significant consequences even for milder situations, potentially catastrophizing instances where their mothers are likely to be angry. This could then increase the likelihood for children to develop internalized aggression for causing their mother's anger. Finally, the impact of maternal anger on children may also depend on whether they can repair their interactions after each anger outburst (Criss et al., 2016). While SECCYD does not provide data to examine such a hypothesis, this hypothesis needs to be tested in future studies.

This study did not observe direct links between fluctuations in maternal depressive symptoms and child depression risks. Although past research identified the fluctuation in maternal depressive symptoms as a risk factor for children, it was conditional on the symptom levels, such that this effect was the most prominent when mothers had high but recovering depression (Wu, 2023). As such, previous research supports that the mean level of maternal depression severity over time may be a more robust predictor of children’s withdrawn depression (e.g., Gartstein et al., 2010; Matijasevich et al., 2015; van Der Waerden et al., 2015; Wu, 2021).

## Theoretical Integration

This study supports life history theory by identifying the fluctuation in maternal anxiety symptoms as a risk factor for children’s development of depression risks, particularly children’s withdrawn depression in middle childhood. This finding was robust beyond the environmental harshness levels (i.e., the mean and the slope of maternal depressive symptoms, anxiety, and anger). This study complements current evidence on life history theory in highlighting maternal mental health (especially anxiety) as a source of unpredictability in shaping children’s development. Findings suggest additional pathways of the intergenerational transmission of depression risks beyond existent literature, in considering fluctuations in anxiety levels over time which can intensify children’s depression risks. Findings also reveal the importance of researching the transdiagnostic feature within the intergenerational transmission of internalizing psychopathology (Doom et al., 2021; Starr et al., 2014) and highlight the importance of considering the comorbidity and co-changes within maternal depression and anxiety.

This study reveals different functions of maternal anxiety and anger in the context of maternal depression. Although both affective expressions can signal emotion dysregulation within depressed individuals (Joormann & Stanton, 2016; Vanderlind et al., 2020), fluctuations in anxiety appeared to intensify the effects of unpredictability on children in the context of maternal depression, as it likely increased maladaptive parenting behaviors. In contrast, variations in anger seem to indicate that some depressed mothers have flexibility in their affect display, thus not increasing children’s risks. Together, it appears that maternal anxiety and anger function in different ways to provide children with exposure to a variety of negative affect, enhancing or inhibiting children’s emotion-related learning. The different effects of maternal anxiety and anger in the context of maternal depression call for further research to unravel their functions in the intergenerational transmission of depression risks.

It is also meaningful to separately examine subtypes of depression-anxiety comorbidity among school-aged children. Separating these subtypes enables researchers and clinicians to identify potential underlying risk factors unique to each presentation, and when such risk factors are known (e.g., high mean maternal depression and anger, and anxiety fluctuations), this information can also aid in risk assessment and prognosis. In addition, it can inform the development of targeted interventions that address the specific cognitive, emotional, and behavioral aspects associated with each subtype. Early intervention strategies can be thus tailored based on the predominant features of anxious or withdrawn depression, potentially mitigating the severity and duration of symptoms.

## Strengths, Limitations, and Clinical Implications

This study has several notable strengths. It complemented the current literature on life history theory by recognizing maternal mental health (especially anxiety) as an unpredictability factor. It examined means, slopes, and fluctuations of maternal factors to separate environmental harshness and unpredictability levels, complementing the largely absent literature in parsing out these effects. It incorporated a large, longitudinal sample to establish robust connections between fluctuations in maternal depressive symptoms and negative affect and child depression risks during a key developmental stage. The study adopted a multi-informant approach with multiple reporters on children. Maternal depressive symptoms, anxiety, and anger were consistently assessed over six years to capture fluctuations during middle childhood.

However, several limitations must be considered. First, the community sample was mostly middle-class, urban, and White, potentially limiting the generalizability of the findings towards samples with elevated risk levels (e.g., samples with clinical levels of depression and/or high SES risks). Second, the study assessed exclusively maternal symptoms, making it challenging to extend the findings to other caregivers such as fathers and grandparents. In addition, given the nature of secondary data, maternal anxiety and anger were measured as “state” affect rather than “trait” affect, and these affective states were not particular to situations involving interactions with their children. Future studies should consider measuring these affective expressions during mother-child interactions. Additionally, given the available data, this study did not examine how children responded to mothers’ affective expressions and their interactions after such expressions, which is a direction of future research. Finally, this study is among the first steps to test whether the degrees of fluctuations in maternal depressive symptoms, anxiety, and anger were linked with depression risks among school-aged children. Nevertheless, considering the dynamic nature of mother-child interactions during development and the potential reciprocal influence between mothers' depression and children's behavioral problems (e.g., Gross et al., 2008; Wu et al., 2019), future studies should consider mother-child mutual influences when exploring the multifaceted associations between fluctuations in maternal depressive symptoms and child depression risks.

In summary, the present study suggests that fluctuations in maternal anxiety, in addition to depressive symptoms, can contribute to an unpredictable developmental environment for children, posing particular challenges for them. The fluctuations in maternal negative affect may have enduring consequences, increasing the risk of children developing depression in middle childhood. The findings offer insights into the importance of ongoing and consistent monitoring and management of maternal depressive symptoms and negative affect through various forms of care, such as counseling, medication, social support, and self-care practices. Additionally, children of mothers with greater anxiety fluctuations might be encouraged to participate in preventative programs aimed at enhancing their regulatory abilities and reducing emotional symptoms. A comprehensive understanding of how and when maternal depressive symptoms impact children's depression risks will guide more tailored interventions for this population, with the potential to foster healthy development in their children.

## Data Availability

The data that support the findings of this study are openly available in the Inter-university Consortium for Political and Social Research at https://www.icpsr.umich.edu/web/ICPSR/series/233.
